# Change in cytokine profiles released by mast cells mediated by lung cancer-derived exosome activation may contribute to cancer-associated coagulation disorders

**DOI:** 10.1186/s12964-023-01110-7

**Published:** 2023-05-04

**Authors:** Suqin Ben, Xiulin Huang, Yongxin Shi, Ziheng Xu, Hui Xiao

**Affiliations:** Department of Respiratory and Critical Care Medicine, Shanghai General Hospital, Shanghai Jiao Tong University School of Medicine, Shanghai, China

**Keywords:** Mast cells, Cancer-associated coagulation disorders, Cancer-associated thrombosis, Exosomes, Lung cancer

## Abstract

**Background:**

Coagulation disorders are a significant cause of lung cancer mortality. Although mast cells are known to play a role in coagulation abnormalities, their specific role in this process has not yet been elucidated.

**Method:**

We detected mast cells in the tumor microenvironment using single-cell sequencing data and examined their correlation with thrombosis-related genes, neutrophil-related genes, neutrophil extracellular trap-related signature genes, and immune infiltration levels in lung cancer patients through bioinformatics analysis. Bone marrow mast cell uptake of exosomes isolated from the lung adenocarcinoma cell line A549, which were labeled using PKH67, was observed using confocal microscopy. Mast cell degranulation was detected by measuring the β-hexosaminidase release rate. Additionally, cytokine array analysis was performed to identify altered mediators released by bone marrow mast cells after uptake of the exosomes.

**Results:**

In our study, we found a close correlation between the proportion of mast cells in lung cancer patients and the expression levels of thrombosis-related genes and neutrophil extracellular trap signature genes, both of which play a key role in thrombophilic disorder. Moreover, we discovered that lung cancer cell-derived exosomes can be taken up by mast cells, which in turn become activated to release procoagulant mediators.

**Conclusion:**

Our study shows that exosomes derived from lung cancer cells can activate mast cells to release procoagulants that may contribute to abnormal blood clotting in lung cancer patients.

Video Abstract

**Supplementary Information:**

The online version contains supplementary material available at 10.1186/s12964-023-01110-7.

## Introduction

Lung cancer remains the leading cause of cancer-related deaths, with approximately 1.8 million deaths per year [1]. Cancer-associated coagulation disorders (CACD), particularly cancer-associated thrombosis (CAT), are a significant complication of cancer patients and are the leading cause of mortality second only to cancer itself [2]. The likelihood of CACD varies among different cancer types, with non-small cell lung cancer posing a significantly higher risk compared to other types [3]. In recent years, there has been an increase in the incidence of lung cancer combined with thrombosis, possibly due to the improved survival of patients resulting from advancements in diagnosis and treatment strategies. Compared to non-cancer patients with thrombosis, those with CAT have a mortality rate that is two to six times higher [4]. As the use of immunotherapies and targeted therapies targeting tumor vasculature or immune checkpoint molecules increases, there is a growing interest in achieving durable normalization of the tumor microenvironment (TME) to improve the efficacy of tumor therapies. Recent studies have highlighted a close link between the coagulum and the TME. Preventing local activation of coagulation in tumors may help to achieve TME normalization [2, 5]. Research on the mechanisms of the hypercoagulable state in lung cancer patients could facilitate the development of new treatment strategies. By gaining a better understanding of this phenomenon, doctors may be able to prevent thrombotic complications and improve the efficacy of cancer treatment.

Mast cells (MCs) are innate immune cells that differentiate from hematopoietic progenitor cells in the bone marrow [6]. While they are well known for their role in allergies, a new understanding has emerged in which they are recognized as a major component of the immune microenvironment of tumors and a source of proinflammatory and angiogenic mediators within TME. MCs are usually located at the margins of tumor tissue, primarily around blood vessels [7]. Upon activation through a stimulus, MCs will degranulate and release inflammatory mediators into the surrounding area. Recently, it has been recognized that inflammation is a common pathway for the formation of blood clots, which can be triggered by various risk factors [8]. Inflammatory mediators likely initiate the coagulation pathway by activating vascular endothelial cells, platelets, and leukocytes [9, 10]. Considering the regulatory role of MCs in the chronic inflammatory response of tumors [11] and their specific localization, we speculate that they may also play a role in CACD.

There is accumulating evidence of crosstalk between coagulation and the immune system. Inappropriate interaction of the immune system and the coagulation system can lead to pathological thrombosis [12]. Within the TME, activated MCs become highly pro-inflammatory and actively recruit immune cells, such as neutrophils and macrophages [7]. Moreover, MC granules contain endothelial cell activators such as histamine and tumor necrosis factor-α (TNF-α). Histamine has been reported to be involved in the mechanism by which MCs exacerbate deep vein thrombosis [13]. TNF-α is known to activate neutrophils and trigger the formation of neutrophil extracellular traps (NETs) [14], which have been extensively explored for their role in the mechanisms of CAT [15]. Thus, activated MCs can have detrimental effects on the vessel wall either directly or indirectly by releasing mediators, which in turn promotes CAT.

Exosomes are small, membrane-bound vesicles with a diameter of 30–100 nm, which can deliver proteins, lipids, and nucleic acids between cells and play key roles in remodeling TME [16]. In our previous study, we found that exosomes derived from lung cancer contain stem cell factor (SCF), which can activate MCs. This activation leads to the release of tryptase, contributing to angiogenesis [17]. The angiogenic cascade can be initiated in several ways by thrombin. When endothelial cells are briefly exposed to thrombin, vascular endothelial growth factor receptors (KDR and Flt-1) are upregulated. These receptors are essential mediators of angiogenesis [18]. These results suggest that MCs within the TME may promote thrombosis. Nevertheless, the specific mechanisms involved have not yet been fully elucidated. Therefore, this study aims to investigate the relationship between MCs and CACD in lung cancer, as well as the underlying mechanisms of their role.

## Methods and materials

### Patients and clinical data collection

We reviewed the medical records of 87 patients who were admitted to Shanghai General Hospital between 2016 and 2022. Based on their medical history, we stratified the enrolled patients into three groups: (a) patients with lung cancer and thrombosis, (b) patients with lung cancer only, and (c) patients with thrombosis but no lung cancer. In all cases, the diagnosis of lung cancer was confirmed through pathology. For cases of lung cancer combined with thrombosis, the diagnosis was only confirmed in individuals whose thrombosis was diagnosed after their lung cancer diagnosis. We retrospectively collected clinical characteristics and plasma biomarkers related to coagulation and tumors from all patients for analysis of similarities and differences. The variables were collected within one month of a lung cancer diagnosis or thrombosis diagnosis. This study was approved by the Ethics Review Board of Shanghai General Hospital (NO.2022KY011, Shanghai, China).

### Bioinformatic analysis

We downloaded the single-cell transcriptome dataset, GSE131907, from the GEO database (https://www.ncbi.nlm.nih.gov/geo/), which included 58 sequences from 44 patients. Next, we selected lung adenocarcinoma (LUAD) tissues for further analysis. After quality control, a total of 42,570 cells were used for subsequent analysis. The data were analyzed with the "Seurat" package to perform Principal Component Analysis. Uniform Manifold Approximation and Projection was used to visualize cell types and clusters [19]. With the Find Clusters function in the Seurat package, we performed integrated clustering of expression values based on shared-nearest-neighbor graph clustering using the Louisville community detection-based method. We evaluated the robustness and further clustering analyses by using the clustering method at different resolutions with different representative markers. In total, we annotated 7 types of cells according to their representative genes, including MCs, T cells, myeloid cells, epithelial cells, B lymphocytes cells, fibroblasts and endothelial cells. The specific annotated marker genes can be found in Supplementary Table 1.

We downloaded the mRNA expression profiles and associated clinical information for 527 LUAD samples and 59 non-LUAD samples from The Cancer Genome Atlas database (TCGA, http://cancergenome.nih.gov/). After normalizing the RNA-seq data to transcripts per million and applying a log_2_ transform, we removed samples with incomplete clinical information for joint analysis of RNA-sequencing and clinical data.

We used the xCell algorithm to estimate the proportion of MCs in the samples from the TCGA data. Then, we sorted all LUAD samples by the proportion of MCs and determined the median value (0.00694578). Subsequently, we divided the LUAD samples into two groups based on their MC proportion: the high MC proportion group and the low MC proportion group. Next, we used CIBERSORT, EPIC, MCP-counter, quanTIseq, TIMER, and xCell algorithms to calculate the infiltration level of immune cells in the TME of LUAD [20–25]. We also used the Wilcoxon test to assess the association between the infiltration level of immune cells and MC proportion, and selected the immune cells with *p* < 0.0001 to visualize them as a heatmap. Additionally, we collected the lists of thrombosis-related signature genes, neutrophil marker genes, and NET signature genes from the reference literature (Supplementary Table 2) [26, 27]. We also obtained the list of 129 immunomodulators (Supplementary Table 3), including chemokines, interleukins, interferons, receptors, and other cytokines, from the study of Charoentong et al. [28]. Subsequently, we analyzed the relationship between the proportion of MCs and the expression levels of these genes and visualized them using the "ggplot2" R package.

### Cell culture

Bone marrow-derived mast cells (BMMCs) were produced by culturing cells extruded from the femurs of 4–6 weeks C57BL/6 mice. Cells were cultured on Roswell Park Memorial Institute 1640 medium (Gibco, USA) containing 10% fetal bovine serum (FBS) (BI, Israel), 10 ng/ml recombinant interleukin-3 (rIL-3) (PeproTech, USA), 5 × 10^-5^ M β-mercaptoethanol (Sigma, USA), 2 mM L-glutamine (Gibco, USA), 10 mM HEPES (Beyotime, China), 10 × nonessential amino acids (Beyotime, China) and 1% penicillin–streptomycin (Beyotime, China). The medium was replaced per week and all cell cultures were grown at 37 °C in a humidified atmosphere with 5% CO_2_. BMMCs with 99% purity were harvested after four weeks of culture and identified by toluidine blue staining, FcεR1, and CD117 expression.

Human LUAD cell line A549 was purchased from Coweldgen Scientific Co., Ltd (Shanghai, China). The cells were cultured in Ham's F-12 K (Kaighn's) medium (BasalMedia, China) with 10% FBS (BI, Israel) and 1% penicillin–streptomycin (Beyotime, China) in a humidified 5% CO_2_ atmosphere at 37 °C.

### Isolation of exosomes

Exosomes were isolated from A549 conditioned media. After the cells reached 90% confluence, the media was centrifuged at 300 g for 10 min, then at 10,000 g for 30 min at 4 °C, and filtered through a 0.22 μm membrane (Merck Millipore, Ireland) to remove cells, debris, and large vesicles. The exosomes were then isolated by ultracentrifugation at 120,000 g for 70 min at 4 °C, washed with phosphate buffer solution (PBS), and centrifuged again at 120,000 g for 70 min at 4 °C. The resulting pellets were resuspended in PBS and stored at -80 °C until use.

### TME of exosomes

We prepared A549-derived exosome samples for transmission electron microscopy (TEM) analysis, as previously described [17]. Briefly, the exosome pellets were loaded onto thin bar carbon-coated copper grids with a 200 mesh and fixed with 2.5% glutaraldehyde. The samples were then washed, contrasted with 2% uranyl acetate, and embedded in a mixture of 0.4% uranyl acetate. Finally, examination was performed using an LEO 912AB Omega electron microscope (Carl Zeiss NTS, Oberkochen, Germany).

### NTA of exosomes

The size distribution and concentration of exosomes diluted in PBS were analyzed by nanoparticle tracking analysis (NTA) using a ZetaVIEW® instrument (Particle Metrix, Germany). The equipment used a 405 nm excitation laser and was pre-calibrated for concentration using a 100 nm polystyrene latex reference standard (Applied Microspheres, Netherlands). The following parameters were used for the detection of exosomes (sensitivity: 85, shutter: 70, minimum brightness: 20, minimum size: 10, maximum size: 200) and the videos were taken at 30 frames per second. The data were analyzed using ZetaView software (Particle Metrix, Germany).

### Confocal microscopy

Isolated exosomes were labeled with PKH67 (Sigma-Aldrich, USA) and then centrifuged again at 120,000 g for 70 min to remove excess dye. The labeled exosomes were co-incubated with BMMCs for 24 h. BMMCs were collected and washed twice with PBS, fixed with 4% formaldehyde for 15 min, and then washed twice with PBS. After labeling the nuclei with DAPI staining solution (Absin, China), the cells were photographed using spinning disc confocol microscopy and Leica SP8 confocal microscopy (Leica, Germany).

### Degranulation assay of β-hexosaminidase release rate

The assay of BMMCs β-hexosaminidase release rate was performed using a previously described method [17], in which BMMCs (5 × 10^5^ cells/ml, 0.5 ml) were prepared for culture in a 24-well plate with three replicate wells per group. The cells were washed twice and resuspended in 500 μl Tyrode's solution. Human SCF (50 ng/ml, PeproTech, USA), different concentrations of exosomes (50μg/well or 100μg/well), and 1μl DNP-IgE (1 mg/ml, Sigma, USA) were added to the different experimental groups for further analysis. The cells were incubated for 24 h, washed with Tyrode's solution, and the IgE group sensitized with DNP-HA (500 ng/well, Sigma, USA) for 45 min. All groups were incubated in an ice bath for 10 min to end the reaction. After incubation, the cell supernatant from each well was collected and centrifuged at 450 g for 5 min. Then, 50 μL of the supernatant was transferred to a separate well of a 96-well plate, followed by the addition of 50 μL of p-nitrophenyl-N-acetyl-beta-D-glucosaminide substrate (Aladdin, China). The plate was incubated at 37 °C for 60 min, and then 200 μL of stop buffer (400 mmol/L glycine, pH 10.4) was added to each well. The absorbance of each well was measured at 405 nm.

After discarding the supernatant, 100 μl of Triton X-100 (1%, Sigma, USA) was added to each well to fully lyse the cells at 37 °C for 30 min, after which the lysate was centrifuged at 10,000 × g for 30 min. Subsequently, the absorbance of the supernatant of the lysate was measured for each sample. The release rate (%) of β-hexosaminidase was calculated by dividing the absorbance of the supernatant by the absorbance of the cell lysate supernatant.

### Cytokine array

We performed a cytokine antibody array using a mouse XL cytokine array kit (Cat. ARY028, R&D Systems, USA) following the instructions of the manufacturer. In brief, after treatment with 50 μg A549-derived exosomes or an equal volume of PBS for 24 h, the supernatant of BMMCs was collected, and the particulates were removed by centrifugation. Diluted supernatants (1:3) and the membranes in the kit were incubated on a rocking platform shaker at 4 °C overnight. After washing, the detection antibody cocktail was added to each membrane for one hour, followed by washing and addition of streptavidin–horseradish peroxidase for 30 min. After another round of washing and addition of Chemi Reagents Mix, immunoblot images were captured using a Tanon system, and the intensity of each spot was analyzed using Image J software.

### Western blotting

Western blotting was performed as previously described. Briefly, exosomes and cell lysates were prepared using RIPA Lysis Buffer (Beyotime, China) supplemented with protease and phosphatase inhibitors (Bimake, USA). Proteins were separated by SDS-PAGE and transferred onto PVDF membranes. The membranes were blocked for 30 min in a blocking solution and incubated overnight at 4 °C with various primary antibodies (anti-TSG101, anti-Calnexin, anti-GAPDH, Abcam, USA; anti-CD81, Santa Cruz Biotech, Germany). After incubation with secondary antibodies for 1 h at room temperature, chemiluminescence detection was performed using the Omni-ECL™ Pico Light Chemiluminescence Kit (Epizyme Biotech, China).

### Statistics

The statistical analysis was performed using SPSS 25.00, R (version 4.1.0), various R packages, and GraphPad Prism 8.0.2. The categorical variables were tested by the chi-square test. For continuous parameters, the t-test is used if the distribution is normal, and the Mann–Whitney U test if it is not normal. For all analyses, we defined statistical significance as *p* < 0.05.

## Results

### Comparison of coagulation-related markers

This study included 17 lung cancer patients with combined thrombosis and 50 lung cancer patients without thrombosis. Patient characteristics are presented in Table 1. There were no significant differences between the two groups. The coagulation indices of the two groups were compared and are presented in Table 2. Prothrombin time (PT), thrombin time (TT), and fibrinogen (FIB) levels did not differ significantly between the two groups. However, levels of fibrin degradation products (FDP) and D-dimer were significantly higher in the thrombus group than in the control group (*p* < 0.001), consistent with previous reports showing positive correlations between FDP, D-dimer, and thrombosis in lung cancer patients [29]. The thrombus group also had a shorter activated partial thromboplastin time (APTT, *p* = 0.036) and higher antithrombin III (AT-III) activity (*p* = 0.017). Moreover, we compared the coagulation indices of 20 non-cancer patients with thrombosis with those of the cancer group. There were no significant differences in TT, FIB, FDP, D-dimer, and AT-III activity, but the PT and APTT were lower in the cancer group than in the non-cancer group (*p* < 0.05) (Table 3).

**Table 1:** Clinical characteristics of patients with lung cancer

		Thrombus(n=17)	Non-thrombus(n=50)	P
Gender				
	Male	6 (35.3%)	24 (48%)	0.363
	Female	11 (64.7%)	26 (52%)	
Age, years				
		65.94 ± 6.088	65.34 ± 12.79	0.599
Smoking history				
	Yes	3 (17.6%)	1 (2%)	0.078
	No	14 (82.4%)	49 (98%)	
Chemotherapy history				
	Yes	15 (88.2%)	35 (70%)	0.242
	No	2 (11.8%)	15 (30%)	
Target therapy				
	Yes	8 (47.1%)	26 (52%)	0.725
	No	9 (52.9%)	24 (48%)	
Immunotherapy				
	Yes	2 (11.8%)	11 (22%)	0.571
	No	15 (88.2%)	39 (78%)	
Histology				
	Squamous carcinoma	2 (11.8%)	10 (20%)	0.025
	Adenocarcinoma	11 (64.7%)	39 (78%)	
	Other	4 (23.5%)	1 (2%)	
Stage				
	I	1 (5.9%)	3 (6%)	0.328
	II	1 (5.9%)	3 (6%)	
	III	5 (29.4%)	5 (10%)	
	IV	10 (58.8%)	39 (78%)	
T				
	1	3 (17.6%)	9 (18%)	0.661
	2	4 (23.5%)	13 (26%)	
	3	4 (23.5%)	5 (10%)	
	4	6 (35.3%)	20 (40%)	
N				
	0	1 (5.9%)	6 (12%)	0.543
	1	1 (5.9%)	7 (14%)	
	2	5 (29.4%)	9 (18%)	
	3	10 (58.8%)	26 (52%)	
Metastasis				
	Yes	8 (50%)	13 (65%)	0.500
	No	8 (50%)	7 (35%)	

Data are expressed as the number of patients (%) or means ± standard deviation. Differences between the two groups were compared using the Chi-square test for categorical variables and Mann-Whitney's U test for the non-normally distributed continuous variable (age)

**Table 2:** Comparison of coagulation parameters

	Thrombus (n=17)	Non-thrombus(n=50)	*P*
PT, s	12.36 ± 1.41	12.87 ± 1.11	0.13
TT, s	17.01 ± 1.37	16.68 ± 1.05	0.3
APTT, s	28.21 ± 4.25	29.40 ± 3.03	0.036
FIB, g/L	4.03 ± 1.88	3.88 ± 1.45	0.983
FDP, mg/L	13.22 ± 10.70	5.18 ± 9.11	0.001
D-Dimer, mg/L	4.03 ± 1.88	1.63 ± 3.35	0
AT-III, %	91.32 ± 10.33	82.33 ± 10.77	0.017

The data are presented as mean ± standard deviation. Non-normally distributed variables, including APTT, FIB, FDP, and D-dimer, were compared using Mann-Whitney tests, while normally distributed variables such as PT, TT, and AT-III were compared using t-tests

*Abbreviations: PT* Prothrombin time, *TT* Thrombin time, *APTT* Activated partial thromboplastin time, *FIB* Fibrinogen, *FDP* Fibrin/fibrinogen degradation products, *AT-III* Antithrombin III

**Table 3:** Coagulation indicators of patients with thrombosis with or without lung cancer

	LC(n=17)	Non-LC(n=20)	*P*
*PT, s*	*12.36 ± 1.41*	*13.91 ± 2.83*	*0.017*
APTT, s	28.21 ± 4.25	37.69 ± 14.11	0.000
TT, s	17.01 ± 1.37	16.87 ± 1.85	0.443
FIB, g/L	4.03 ± 1.88	4.45 ± 1.81	0.641
FDP, mg/L	13.22 ± 10.70	31.23 ± 33.72	0.182
D-Dimer, mg/L	6.8 ± 7.18	9.31 ± 10.99	0.537
AT-III, %	91.32 ± 10.33	87.93 ± 12.1	0.519

All data were present as mean ± standard deviation. Mann–Whitney test was used to compare the differences

*Abbreviations:* *LC* Lung cancer, *PT* Prothrombin time, *TT* Thrombin time, *APTT* Activated partial thromboplastin time, *FIB* Fibrinogen, *FDP* Fibrin/fibrinogen degradation products, *AT-III* Antithrombin III

### Bioinformatics analysis of the association between MCs and CAT

**Fig. 1:**
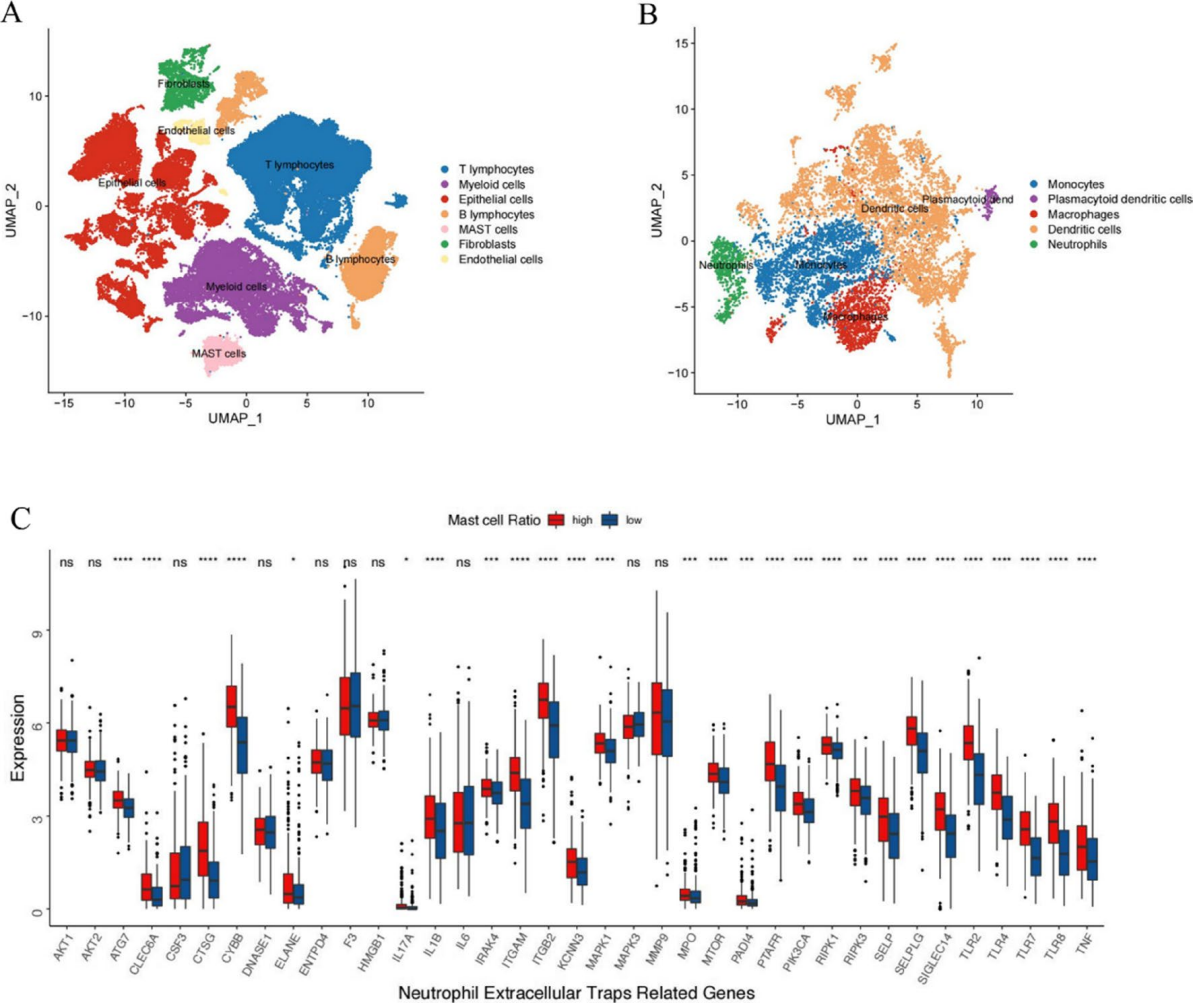
Bioinformatics analysis of the correlation between mast cells and cancer-associated thrombosis. **A, B** Cluster diagram of different cell types. **C** Expression of neutrophil extracellular traps signatures in different proportions of mast cells in lung cancer samples

We first analyzed the single-cell sequencing data of LUAD, selecting 42,570 cell sequences from LUAD samples for further analysis. Cells were clustered by representative marker genes from reference literature, revealing the presence of MCs in the TME of lung cancer (Fig. 1A). Tumor-infiltrating myeloid cells, including monocytes, macrophages, neutrophils, dendritic cells, and plasma dendritic cells, were further distinguished (Fig. 1B). We then examined the relationship between the proportion of MCs and thrombosis in lung cancer. Based on the median of MC proportion, we divided LUAD patients into two groups with high and low proportions of MCs. Using 140 thrombosis-associated genes, we compared the differential expression of these genes in the two groups and found that 92 genes related to thrombosis had significantly different expressions (*p* < 0.05, data not shown). Because NETs play a crucial role in the CAT, we identified typical marker genes of NETs from literature [27] and observed that the high MC group exhibited elevated levels of NETs formation (Fig. 1C). Additionally, given the emerging evidence for a strong link between thrombosis and immune cells, we also investigated the association between MC proportion and the expression levels of common tumor-associated regulatory molecules, as well as immune cell infiltration. As shown in Supplementary Fig. 1A, the high proportion of MCs in the lung cancer group was significantly correlated with increased levels of immune cell infiltration, including monocytes, neutrophils, and macrophages. Additionally, there were significant differences in the expression levels of tumor-associated regulatory molecules between the high and low MC groups, as demonstrated in Supplementary Fig. 1B.

### Identification of BMMCs and exosomes

**Fig. 2:**
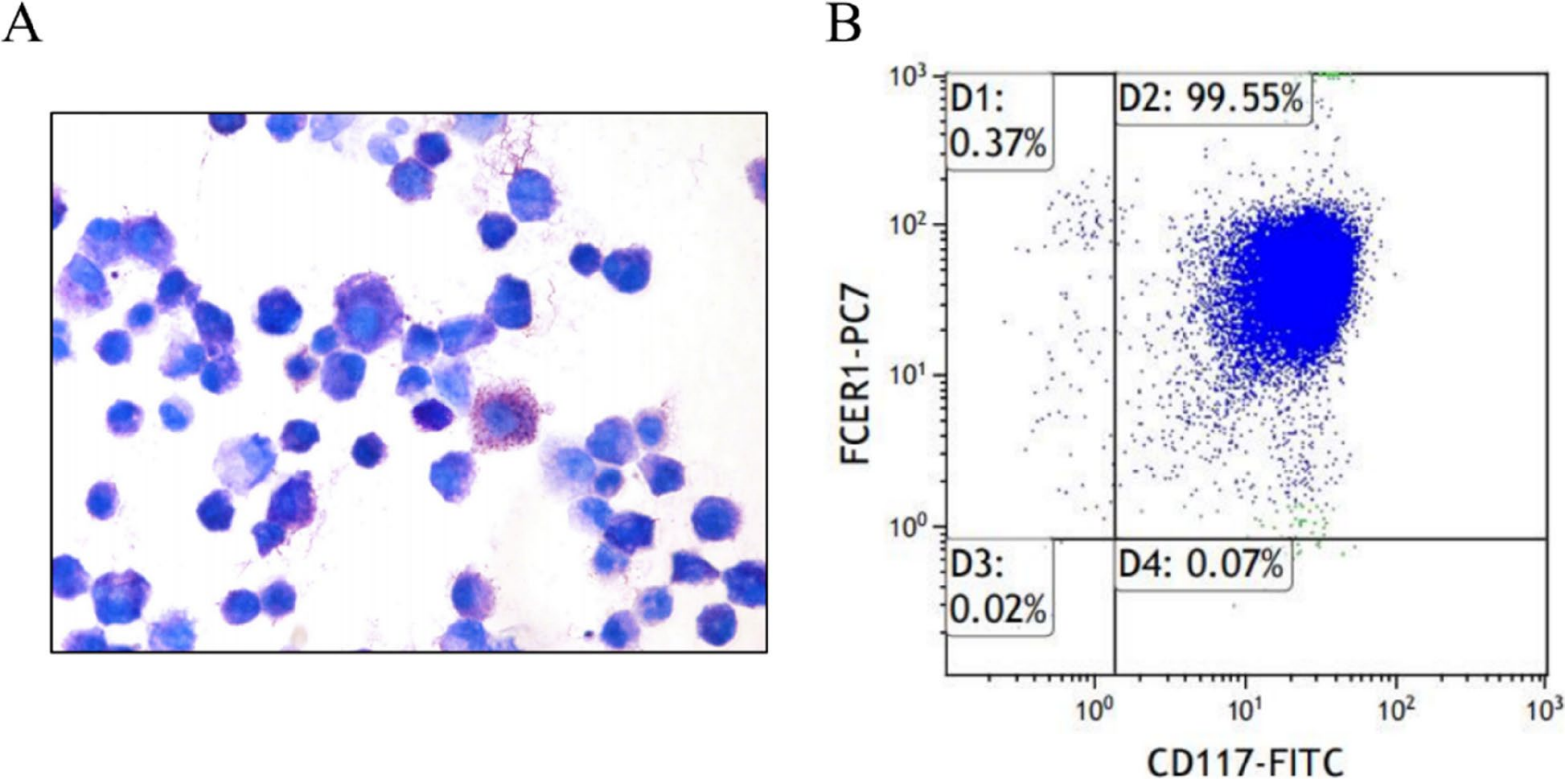
The characteristics of bone marrow mast cells. **A** Toluidine blue staining for bone marrow mast cells (BMMCs). Magnification, × 400. **B** Flow cytometry detection of FcεR1 and CD117 expression in BMMCs

After 4 weeks of culture in an IL-3-containing medium, bone marrow-derived cells differentiated into MCs, as confirmed by staining with toluidine blue (Fig. 2A). The cytoplasm of BMMCs contained a large number of purple granules. To further confirm the purity of the induced MCs, we performed flow cytometry analysis based on the expression of IgE receptor (FcεR1) and CD117, which showed a purity of more than 99.55% (Fig. 2B).

**Fig. 3:**
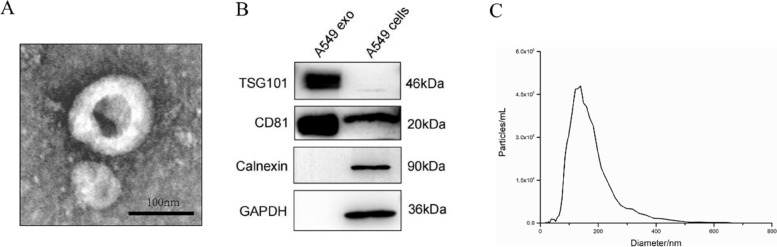
Characterization of exosomes derived from A549 cells. **A** Transmission electron micrograph of A549-derived exosomes. Scale bar = 100 nm.** B** Representative Western blot images for exosome biomarkers. **C** Nanoparticle tracking analysis of exosomes from A549

The exosomes derived from A549 cells were extensively characterized using TEM, NTA, and Western blotting. TEM imaging revealed the presence of small, round, vesicular structures typical of exosomes (Fig. 3A). NTA analysis further confirmed an average particle size of approximately 130 nm (Fig. 3C). Western blot analysis demonstrated high levels of exosomal markers CD81 and TSG101 in the exosome pellets compared to the cell lysates (Fig. 3B). Importantly, the exosome lysates did not show any detectable expression of Calnexin, a negative exosomal marker. Collectively, these results confirm that the isolated exosomes meet the established criteria for downstream experiments.

### Uptake of exosomes by BMMCs and the effect on them

**Fig. 4:**
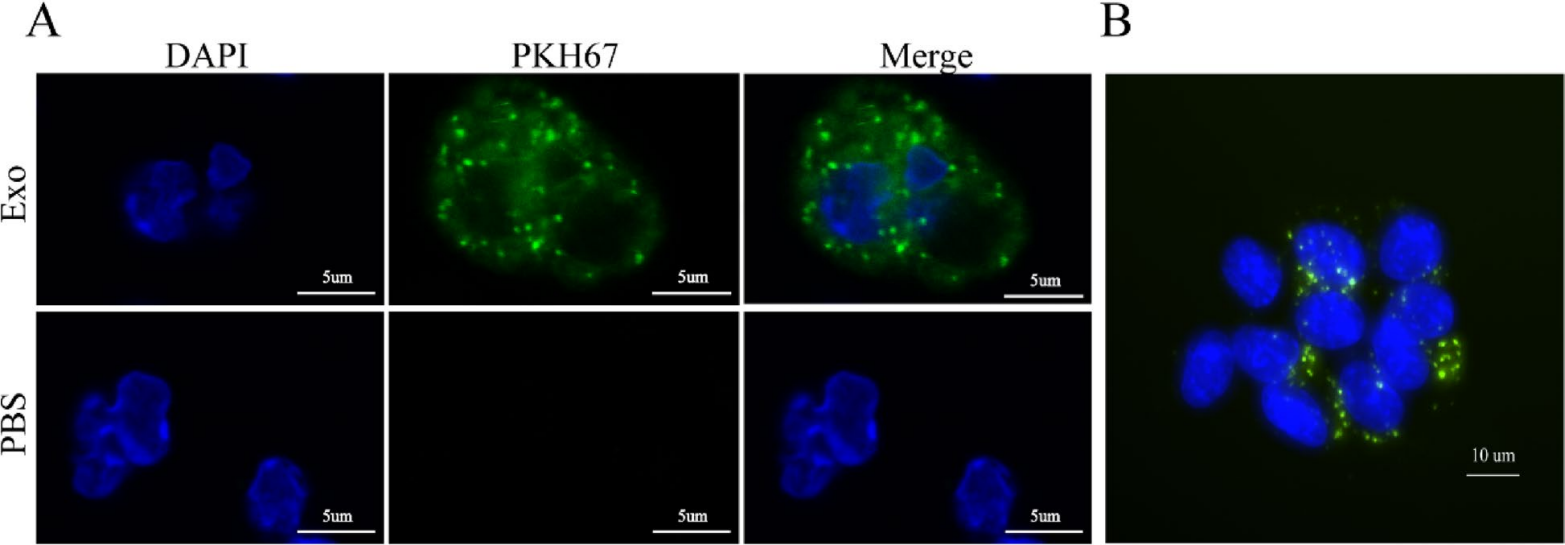
Uptake of exosomes from A549 cells by BMMCs. **A** and **B** Representative confocal microscopy images of mast cells taking up PKH67-labeled exosomes. (Green: PKH67-stained exosomes, Blue: DAPI nuclear stain)

**Fig. 5:**
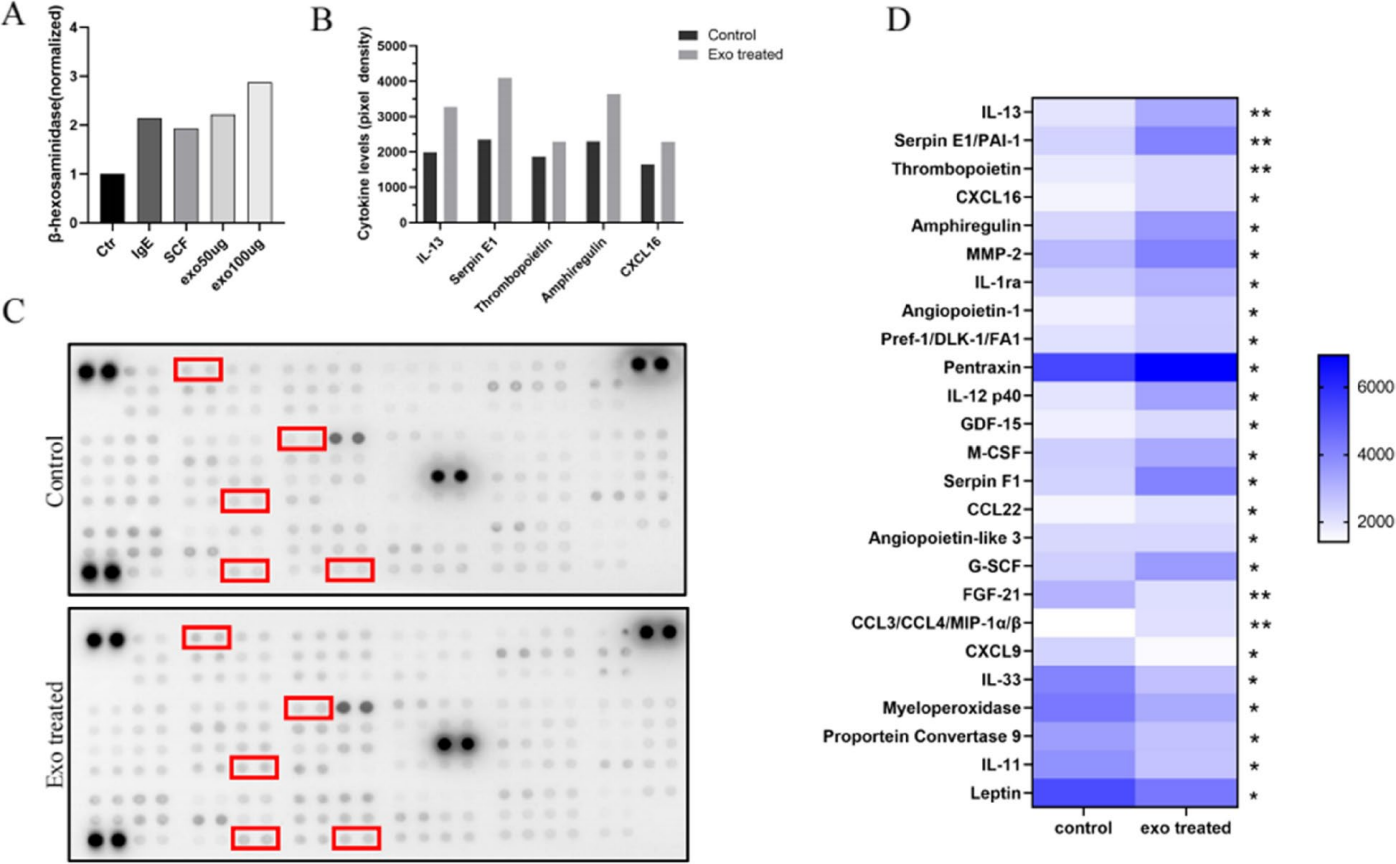
Degranulation assay and cell supernatant cytokine levels after mast cell uptake of A549-derived exosomes. **A** Detection of mast cell degranulation after different treatments. **B** Quantitative grayscale analysis of cytokine levels. **C** Cytokine array analysis.** D** The heat map demonstrates the differential expression of cytokines between the two groups. *P ≤ 0.05, **P < 0.01

In a previous study, we demonstrated that lung cancer cell-derived exosomes can stimulate angiogenesis by activating MCs to release mediators [17]. In this study, we observed the uptake of PKH67-labeled exosomes by BMMCs after co-incubation for 24 h (Fig. 4A, B). We used SCF and IgE as positive controls since they are known to activate MCs and induce degranulation. As shown in Fig. 5A, there was a significant dose-dependent increase in MC β-hexosaminidase release after 24 h of incubation with A549 cell-derived exosomes. We then investigated the cytokines released by MCs after exosome uptake. We found that the expression levels of IL-13, SerpinE1, Thrombopoietin, and CXCL16 were significantly higher in MCs after 24 h of incubation with exosomes compared to the control group (Fig. 5B-D).

## Discussion

In this study, we investigated the possible role of MCs in promoting thrombosis in patients with lung cancer by observing changes in the cytokine profile released from MCs after stimulation with lung cancer cell-derived exosomes. Lung cancer is known to offen trigger a hypercoagulable state, leading to thrombotic events and poor prognosis [30]. High levels of D-dimer are considered to be a good predictor of poor prognosis and thrombotic complications in patients with lung cancer [31, 32]. Several studies have reported elevated levels of D-dimer and FDP in lung cancer patients [31, 33]. In this study, we found that lung cancer patients with thrombosis had significantly increased levels of D-dimer and FDP, and a shortened APTT. Furthermore, patients with lung cancer-related thrombosis had a higher procoagulant capacity and exhibited significantly shorter PT and APTT compared to those with thrombotic disease alone. These findings suggest a strong association between coagulation abnormalities and the high incidence of thrombosis in lung cancer patients, and have motivated us to further investigate the underlying mechanisms of coagulation abnormality in this population.

We analyzed 140 thrombosis-associated genes from references to investigate whether they were correlated with the proportion of MCs in lung cancer samples. As expected, we found that most thrombosis-related signatures were differentially expressed in the two groups of samples. The interaction between the coagulation system and the TME has been the focus of many recent studies. Tumor-infiltrating myeloid cells, such as MCs, neutrophils, macrophages, and monocytes, play a role in supporting tumor development at distinct stages. In this study, we used scRNA-seq analysis to identify the presence of these cells in the TME of LUAD. The role of myeloid cells in thromboembolism has gained considerable attention in recent years [34]. Among these cells, MCs are mainly localized at the edge of the tumor tissue, particularly around blood vessels, and are involved in the remodeling of the TME through direct interaction with other cells or by degranulation and release of mediators [7]. MCs contain granules that are rich in proinflammatory mediators, such as TNF-α and histamine, which mediate endothelial activation and cell recruitment, a key event in thrombus formation [35]. Previous studies by Bankl et al. have shown that MCs aggregate in the thrombosed vein and exhibit profibrinolytic properties [36]. However, recent studies suggest that MCs may have the opposite role in thrombosis. For instance, Ponomaryov et al. reported that MC deficiency in mice prevents deep vein thrombosis [13]. Additionally, MCs contain polyphosphates, which have been shown to have a powerful procoagulant effect [37]. Therefore, the precise role of MCs in CACD remains to be fully elucidated.

In CAT, neutrophils are among the first immune cells to arrive at the site of vascular damage [38]. Once activated, they can form NETs, which act as a scaffold for procoagulants and are closely associated with thrombosis in cancer [15]. Sharma et al. investigated the role of NETs in chronic thrombosis and found that the expression of markers of neutrophil activation was significantly increased in patients with this condition [39]. Interestingly, we observed that patients with a high proportion of MCs had significantly higher expression of NETs markers than those with a low proportion of MCs. This suggests that MCs may play a role in modulating the activity of neutrophils and the formation of NETs in CAT.

Tumor-associated MCs are known to release tryptase, which has been shown to significantly impact the formation of NETs [40]. Additionally, MCs can release extracellular traps and contribute to the evolution of coronary thrombosis [41]. Given the close association between thrombosis and immune cells in the TME, we analyzed the correlation between MC proportion and immune cell infiltration using multiple algorithms. Previous studies have shown that innate immune cells, particularly monocytes, neutrophils, and dendritic cells, promote fibrin formation and trigger platelet activation during thrombosis [42]. The activation of coagulation also promotes the subsequent recruitment of myeloid cells. Consistent with these findings, we observed higher infiltration levels of these cells in the group with a high proportion of MCs. Based on previous research and our bioinformatics analysis, we hypothesize that MCs may be involved in the mechanism of CACD.

The role of MCs in the mechanism of CACD is not yet fully understood, and to explore their potential contribution, we conducted further experiments. Our findings revealed that MCs can uptake exosomes released by lung cancer cells, leading to degranulation within 24 h (Figs. 4 and 5A). This result aligns with our previous research demonstrating that lung cancer-derived exosomes contain  SCF and modulate MC activity [17]. MCs have a complex biology that has been extensively documented, with the ability to exert direct or indirect effects on pathogens through a diverse array of membrane receptors, and variations in the composition of their cytoplasmic granules and released substances [43]. According to Simonowski et al., KIT employs a distinct mechanism to activate MCs, unlike the conventional activation through FcεRI [44]. We speculate that the mediators released by MCs under the activation of the SCF-KIT signaling pathway will differ from those under other activation pathways. Therefore, we designed a study to specifically examine the mediators released during MC degranulation under SCF-KIT activation, as we had not previously investigated changes in the expression profile of cytokines released by MCs following exosome uptake. To accomplish this, we utilized the Cytokine Array Kit to observe any alterations in the cytokine release profile of MCs (Fig. 5). IL-13, SerpinE1, and Thrombopoietin showed the most significant differences in expression. SerpinE1 has been implicated in thrombosis in various diseases, including tumor-related thrombosis [45]. As a serine protease, SerpinE1 inhibits tissue plasminogen activator and urokinase, which are activators of plasminogen and fibrinolysis, respectively. Previous studies have demonstrated the involvement of SerpinE1 in thrombosis associated with anti-angiogenic therapy [46]. Therefore, it is possible that the increased release of SerpinE1 from MCs triggered by lung cancer-derived exosomes plays a role in CAT.

Thrombopoietin has been reported to induce thrombocytosis and thrombosis in a mouse study [47], with platelets playing a vital role in thrombosis by interacting with cancer cells to promote their activation and aggregation. Elevated levels of activated platelets are involved in the pathogenesis of cancer thrombosis. Additionally, interleukin 13 is closely associated with vascular inflammation, and several studies have shown that inflammation-related cytokines are linked to thrombosis [48].

In conclusion, our results suggest that MCs may contribute to lung cancer-related thrombosis through the release of cytokines, including IL-13, SerpinE1, and Thrombopoietin, following the uptake of lung cancer cell-derived exosomes. These findings provide new insights into the mechanisms underlying CACD and may offer potential targets for therapeutic intervention. However, further studies are needed to confirm our observations and fully elucidate the role of MCs in lung cancer thrombosis.

There are several limitations to this study that should be addressed in future research. Firstly, the effects of cytokines released from MCs on lung cancer combined with thrombosis have not been validated. Further studies should investigate the specific role of these cytokines in the pathogenesis of lung cancer-related thrombosis. Secondly, the mechanisms by which cytokines released from MCs induce thrombosis remain unclear and require further exploration. In future studies, it would be useful to examine the differences in cytokine profiles between SCF-KIT and IgE-FcεRI-induced MC degranulation. Addressing these limitations will help to deepen our understanding of the role of MCs in lung cancer-related thrombosis.

## Conclusions

**Fig. 6:**
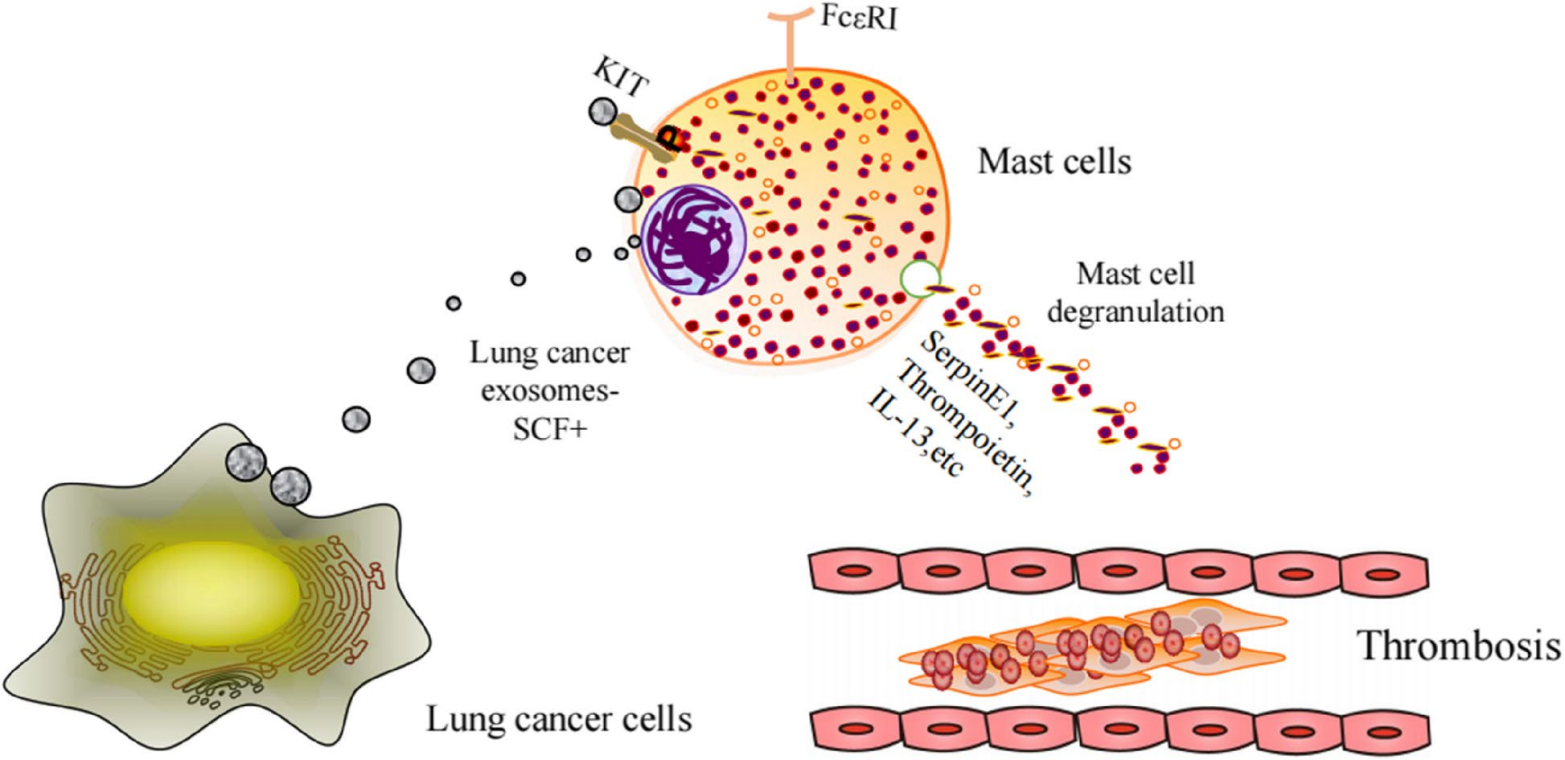
Working model. Mast cells uptake lung cancer-derived exosomes and release SerpinE1, Thrombopoietin, IL-13 and other pro-inflammatory, pro-thrombotic substances

Our study reveals the changes in cytokine expression profiles that occur following activation of MCs by lung cancer cell-derived exosomes and their potential role in lung cancer-associated coagulation disorders (Fig. 6).

## Supplementary Information

**Additional file 1: Supplementary Fig. 1.** Levels of immune cell infiltration, expression of tumor regulatory moleculesin lung cancer patients with different mast cell proportions.

**Additional file 2: Supplementary Table 1.**

**Additional file 3: Supplementary Table 2.** Thrombosis associated gene list.

**Additional file 4: Supplementary Table 3.** List of 129 immunomodulators.

## Data Availability

The datasets analyzed for this study can be found in the TCGA database (https://www.cancer.gov/tcga) and the GEO database (https://www.ncbi.nlm.nih.gov/geo/).

## References

[1] Sung H (2021). Global cancer statistics 2020: Globocan estimates of incidence and mortality worldwide for 36 cancers in 185 countries. CA Cancer J Clin.

[2] Kim AS (2020). Mechanisms and biomarkers of cancer-associated thrombosis. Transl Res.

[3] Tagalakis V (2007). High risk of deep vein thrombosis in patients with non-small cell lung cancer: a cohort study of 493 patients. J Thorac Oncol.

[4] Meikle CK (2020). Platelet-T cell aggregates in lung cancer patients: implications for thrombosis. PLoS ONE.

[5] Galmiche A (2022). Coagulome and the tumor microenvironment: an actionable interplay. Trends Cancer.

[6] Varricchi (2019). Future needs in mast cell biology. Int J Mol Sci.

[7] Komi DEA (2020). Role of mast cells in shaping the tumor microenvironment. Clin Rev Allergy Immunol.

[8] Branchford BR (2018). The role of inflammation in venous thromboembolism. Front Pediatr.

[9] Mahemuti A (2012). Association of interleukin-6 and C-reactive protein genetic polymorphisms levels with venous thromboembolism. Chin Med J.

[10] Matos MF (2011). The role of IL-6, IL-8 and MCP-1 and their promoter polymorphisms IL-6 -174GC, IL-8 -251AT and MCP-1 -2518AG in the risk of venous thromboembolism: a case-control study. Thromb Res.

[11] Aller MA (2019). Carcinogenesis: the cancer cell-mast cell connection. Inflamm Res.

[12] Colling ME (2021). Inflammation, infection and venous thromboembolism. Circ Res.

[13] Ponomaryov T (2017). Mast cells granular contents are crucial for deep vein thrombosis in mice. Circ Res.

[14] Pang L (2013). Pseudogout-associated inflammatory calcium pyrophosphate dihydrate microcrystals induce formation of neutrophil extracellular traps. J Immunol.

[15] Thålin C (2019). Neutrophil extracellular traps: villains and targets in arterial, venous, and cancer-associated thrombosis. Arterioscler Thromb Vasc Biol.

[16] Mashouri L (2019). Exosomes: composition, biogenesis, and mechanisms in cancer metastasis and drug resistance. Mol Cancer.

[17] Xiao H (2019). The release of tryptase from mast cells promote tumor cell metastasis via exosomes. BMC Cancer.

[18] Maragoudakis ME (2000). Effects of thrombin/thrombosis in angiogenesis and tumour progression. Matrix Biol.

[19] Dinh HQ (2021). Integrated single-cell transcriptome analysis reveals heterogeneity of esophageal squamous cell carcinoma microenvironment. Nat Commun.

[20] Racle J (2020). Epic: a tool to estimate the proportions of different cell types from bulk gene expression data. Methods Mol Biol.

[21] Newman A (2015). Robust enumeration of cell subsets from tissue expression profiles. Nat Methods..

[22] Becht E (2016). Estimating the population abundance of tissue-infiltrating immune and stromal cell populations using gene expression. Genome Biol.

[23] Finotello F (2019). Molecular and pharmacological modulators of the tumor immune contexture revealed by deconvolution of Rna-Seq Data. Genome Med.

[24] Li B (2016). Comprehensive analyses of tumor immunity: implications for cancer immunotherapy. Genome Biol.

[25] Aran D (2017). Xcell: digitally portraying the tissue cellular heterogeneity landscape. Genome Biol.

[26] Zeng WJ (2022). A novel thrombosis-related signature for predicting survival and drug compounds in glioblastoma. J Oncol.

[27] Zhang Y, et al. A signature for pan-cancer prognosis based on neutrophil extracellular traps. J Immunother Cancer. 2022;10:e004210. 10.1136/jitc-2021-004210 PMC9189842 35688556

[28] Charoentong P (2017). Pan-cancer immunogenomic analyses reveal genotype-immunophenotype relationships and predictors of response to checkpoint blockade. Cell Rep.

[29] Cui YQ (2021). Analysis on risk factors of lung cancer complicated with pulmonary embolism. Clin Respir J.

[30] Bayleyegn B, et al. Coagulation parameters in lung cancer patients: a systematic review and meta-analysis. J Clin Lab Anal. 2022;36:e24550. 10.1002/jcla.24550 PMC9279983 35719003

[31] Fei X (2017). Tissue factor pathway inhibitor-1 is a valuable marker for the prediction of deep venous thrombosis and tumor metastasis in patients with lung cancer. Biomed Res Int.

[32] Ma M (2021). The D-Dimer level predicts the prognosis in patients with lung cancer: a systematic review and meta-analysis. J Cardiothorac Surg.

[33] Tian B (2018). The significance of perioperative coagulation and fibrinolysis related parameters after lung surgery for predicting venous thromboembolism: a prospective. Single Center Study J Thorac Dis.

[34] Campos J, et al. The role of bone marrow-derived cells in venous thromboembolism. Int J Biochem Cell Biol. 2020;128:105850. 10.1016/j.biocel.2020.105850 PMC7607213 32950686

[35] Budnik I (2018). Immune factors in deep vein thrombosis initiation. Trends Immunol.

[36] Bankl HC (1999). Mast cells are augmented in deep vein thrombosis and express a profibrinolytic phenotype. Hum Pathol.

[37] Mailer RKW, et al. Polyphosphate as a target for interference with inflammation and thrombosis. Front Med. 2019;6:76. 10.3389/fmed.2019.00076 PMC6499166 31106204

[38] Darbousset R (2012). Tissue factor-positive neutrophils bind to injured endothelial wall and initiate thrombus formation. Blood.

[39] Sharma S (2021). Neutrophil extracellular traps promote fibrous vascular occlusions in chronic thrombosis. Blood.

[40] Pejler G (2022). Mast cell tryptase potentiates neutrophil extracellular trap formation. J Innate Immun.

[41] Pertiwi KR (2019). Extracellular traps derived from macrophages, mast cells, eosinophils and neutrophils are generated in a time-dependent manner during atherothrombosis. J Pathol.

[42] Engelmann B (2012). Thrombosis as an Intravascular Effector of Innate Immunity. Nat Rev Immunol.

[43] González-de-Olano D (2018). Mast cells as key players in allergy and inflammation. J Investig Allergol Clin Immunol.

[44] Simonowski A (2020). Differential use of BTK and PLC in FcεRI- and KIT-mediated mast cell activation: a marginal role of BTK upon KIT activation. Biochim Biophys Acta Mol Cell Res.

[45] Hisada Y (2021). Plasminogen activator inhibitor 1 and venous thrombosis in pancreatic cancer. Blood Adv.

[46] Chen N (2015). Bevacizumab promotes venous thromboembolism through the Induction of Pai-1 in a mouse xenograft model of human lung carcinoma. Mol Cancer.

[47] Hisada Y (2015). Venous thrombosis and cancer: from mouse models to clinical trials. J Thromb Haemost.

[48] Saghazadeh A (2016). Inflammation as a cause of venous thromboembolism. Crit Rev Oncol Hematol.

