# Supplementary material for: Change in cytokine profiles released by mast cells mediated by lung cancer-derived exosome activation may contribute to cancer-associated coagulation disorders
Source: Cell Commun Signal. 2023 May 4;21:97. doi: 10.1186/s12964-023-01110-7 (PMC10161433; doi:10.1186/s12964-023-01110-7)
Supplement: Supplementary file 2 — Additional file 1: Supplementary Fig. 1. Levels of immune cell infiltration, expression of tumor regulatory moleculesin lung cancer patients with different mast cell proportions. [file 12964_2023_1110_MOESM1_ESM.docx]

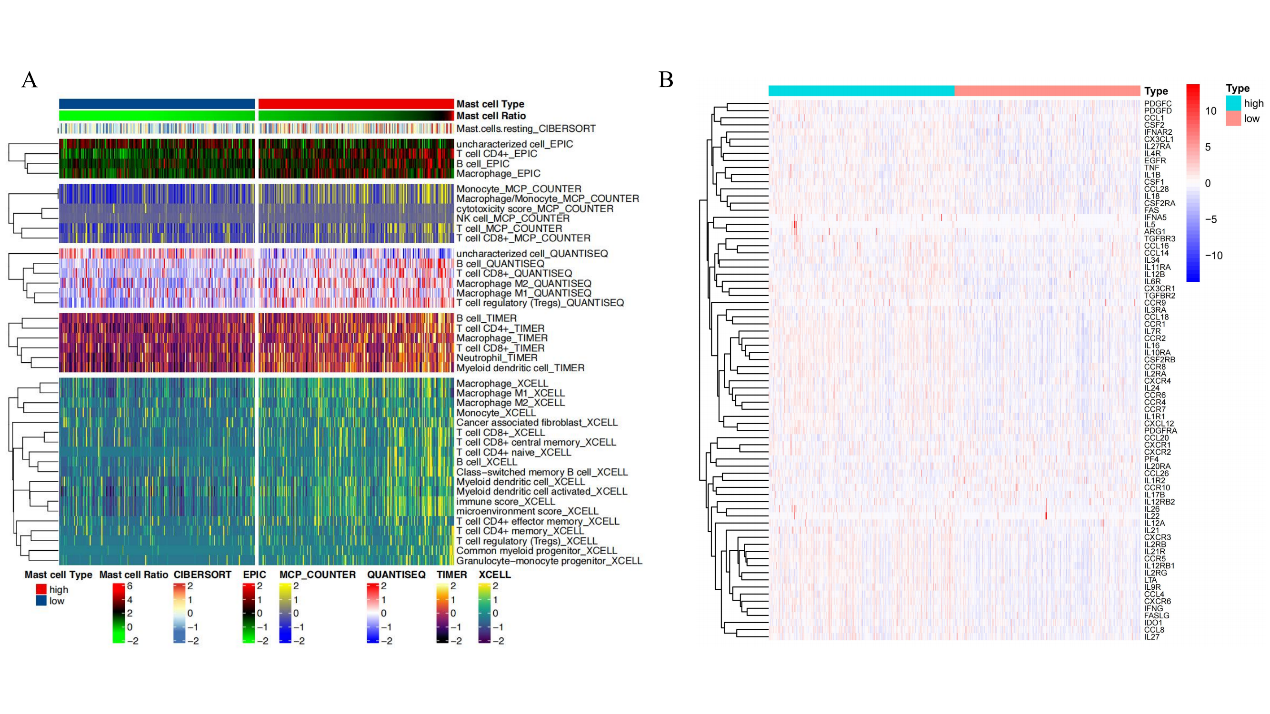
 **Supplementary Fig.1** Levels of immune cell infiltration (A), expression of tumor regulatory molecules (B) in lung cancer patients with different mast cell proportions.

S
